# Draft genome sequence of *Paenibacillus* sp. M.A.Huq-81 isolated from the soil sample of a pine garden located in Anseong, South Korea

**DOI:** 10.1128/mra.01191-25

**Published:** 2025-12-16

**Authors:** Md. Amdadul Huq, Jong-Whi Park, Md. Shahedur Rahman

**Affiliations:** 1 Department of Life Sciences, College of BioNano Technology, Gachon University 65440, Seongnam, Republic of Korea; 2 Department of Genetic Engineering and Biotechnology, Jashore University of Science and Technology 421984, Jashore, Bangladesh; DOE Joint Genome Institute, Berkeley, California, USA

**Keywords:** draft genome sequence, *Paenibacillus *sp. M.A.Huq-81, soil sample of a pine garden

## Abstract

This study reports the draft genome sequence of *Paenibacillus* sp. M.A.Huq-81, a bacterial strain isolated from the soil sample of a pine garden located in Anseong, South Korea, in 2021. The genome consists of 5,210,425 base pairs assembled into 22 contigs, encoding 4,797 predicted protein-coding genes.

## ANNOUNCEMENT

The genus *Paenibacillus*, first identified by Ash et al. (1), is classified under the family *Paenibacillaceae*. To date, it includes 323 validly published species (2) that have been isolated from a wide range of environments such as soil, rhizosphere, water, air, food, milk, human blood, and feces (3–5). During investigations on bacterial biodiversity in the soil sample of a pine garden, a new strain of the genus *Paenibacillus*, designated *Paenibacillus* sp. M.A.Huq-81 was isolated, and its draft genome assembly is presented in this study.

Strain M.A.Huq-81 was isolated from the soil sample of a pine garden, located in Anseong, South Korea (37° 03′ 16″ N, 127° 30′ 59″ E). One gram of soil sample (collection date: February 10, 2021) was suspended in 9 mL of sterile 0.85% (wt/vol) NaCl solution. The suspension was serially diluted up to a 10^−6^ dilution, and 100 μL of individual dilution was spread onto Tryptic Soy Agar (TSA) plates (6). Then, the plates were placed at 28 °C in an incubator for 3 days. Single colonies were purified by repeated streaking on fresh TSA plates (7). The strain M.A.Huq-81 has been deposited into the China General Microbiological Culture Collection Center with the deposition number CGMCC 1.60092. The genomic DNA was extracted from the bacterial culture that was grown in TSB for 24 h using the Solg Genomic DNA Prep kit (Solgent, Republic of Korea), according to the manufacturer’s protocol. The DNA fragments were size selected using AMPure XP beads to isolate those within the desired range, typically 200–300 base pairs (bp) for standard Illumina sequencing libraries. The library for sequencing was prepared using the Nextera XT DNA Library Prep Kit (Illumina, San Diego, USA). After library preparation, sequencing was carried out using the Illumina HiSeq 3000 platform with paired-end 2 × 150 bp reads following the standard protocol provided by the manufacturer. Illumina’s bcl2fastq software version 2.20.0 was utilized with default parameters to demultiplex the raw reads and convert them into FASTQ format. For further downstream analysis, raw paired-end reads from the HiSeq platform were used. Reads were processed with Trimmomatic version 0.38 (8). The genome sequence was assembled by SOAPdenovo v2.04 assembler (9). The genome was annotated with the NCBI Prokaryotic Genome Annotation Pipeline (PGAP) version 6.3 (10–12). The strain was identified using a whole genome-based taxonomic analysis in the Type (Strain) Genome Server (https://tygs.dsmz.de). The genome-to-genome distance (GGDH) bioinformatics tool (13) was used to measure *in silico* DNA-DNA hybridization (isDDH) values. The best matches to the strain M.A.Huq-81 genome are the type strain *Paenibacillus pinistramenti* ASL46 (accession GCA_005869875.1) with *in silico* DNA-DNA hybridization values of 22.4%.

The number of reads generated by genome sequencing was 3,163,908. The genome of *Paenibacillus* sp. M.A.Huq-81 is 5,210,425 bp long with a GC content of 48.0%. The assembly contains 22 contigs with a coverage of 79×. A total of 4,913 genes were predicted, of which 4,797 are protein-coding genes. The details of genomic features are presented in Table 1.

**TABLE 1:** Genomic features of *Paenibacillus* sp. M.A.Huq-81

Feature	Result
Description of source	
Location	Anseong, South Korea
Time	2021
Type	Soil sample of pine garden
Sequencing summary	
Coverage	79×
Total bases	5,210,425
GC content	48.0%
Assembly report	
Contigs	22
Contig *L*_50_	3
Contig *N*_50_	566.4 kb
Genome length	5.2 Mb
Annotation report	
Genes (total)	4,913
CDSs (total)	4,822
CDSs (with protein)	4,797
ncRNAs	4
tRNA	80
Quality analysis	
Completeness	99.17%
Contamination	0.3%

## Data Availability

The draft genome sequence of *Paenibacillus* sp. M.A.Huq-81 was deposited in GenBank under accession number JBRFIM000000000. The raw reads are available in SRA under accession number SRR33604962. The version described in this paper is the first version, JBRFIM000000000.1. The data have been deposited in GenBank under BioSample number SAMN51699928 and BioProject number PRJNA1331960. The genome assembly is available under the accession GCA_052931865.1.
